# Tungiasis Stigma and Control Practices in a Hyperendemic Region in Northeastern Uganda

**DOI:** 10.3390/tropicalmed8040206

**Published:** 2023-03-30

**Authors:** Hannah McNeilly, Marlene Thielecke, Francis Mutebi, Mike Banalyaki, Felix Reichert, Susanne Wiese, Hermann Feldmeier

**Affiliations:** 1 Edinburgh Medical School: Biomedical Sciences, The University of Edinburgh, Edinburgh EH8 9YL, UK; 2 Charité Center for Global Health, Institute of International Health, Charité University Medicine, 13353 Berlin, Germany; 3 School of Veterinary Medicine and Animal Resources, College of Veterinary Medicine, Animal Resources and Biosecurity, Makerere University, Kampala P.O. Box 7062, Uganda; 4 Innovations for Tropical Disease Elimination (IFOTRODE), Kampala P.O. Box 24461, Uganda; 5 Department of Infectious Disease Epidemiology, Robert Koch Institute, 13353 Berlin, Germany; 6 Institute of Microbiology, Infectious Diseases and Immunology, Charité University Medicine, 12203 Berlin, Germany

**Keywords:** tungiasis, stigma, neglected tropical diseases, control practices, hygiene, KAP, Uganda

## Abstract

Neglected tropical diseases are known to be highly stigmatized conditions. This study investigates tungiasis-related stigma and control practices in the impoverished Napak District in rural northeastern Uganda, where tungiasis is hyperendemic and effective treatment is unavailable. We conducted a questionnaire survey with the main household caretakers (*n* = 1329) in 17 villages and examined them for tungiasis. The prevalence of tungiasis among our respondents was 61.0%. Questionnaire responses showed that tungiasis was perceived as a potentially serious and debilitating condition and that tungiasis-related stigma and embarrassment were common. Among the respondents, 42.0% expressed judging attitudes, associating tungiasis with laziness, carelessness, and dirtiness, and 36.3% showed compassionate attitudes towards people with tungiasis. Questionnaire responses further indicated that people made an effort to keep their feet and house floors clean (important tungiasis prevention measures), but lack of water was a common problem in the area. The most frequent local treatment practices were hazardous manual extraction of sand fleas with sharp instruments and application of various and sometimes toxic substances. Reliable access to safe and effective treatment and water are therefore key to reducing the need for dangerous treatment attempts and breaking the vicious cycle of tungiasis stigma in this setting marked by poverty.

## 1. Introduction

Tungiasis is a neglected parasitic skin disease caused by the sand flea *Tunga penetrans*. It is widespread throughout sub-Saharan Africa and South America, particularly among the most marginalized populations [1,2]. Female sand fleas burrow into the skin of humans and animals, usually in the feet, where they grow and shed eggs onto the ground [1,3]. A risk factor analysis from Kenya identified regular washing of the feet with soap as well as frequent cleaning of house floors as protective factors, and consequently, lack of access to water and soap as a risk factor for tungiasis [4]. To date, the only safe tungiasis treatment proven to kill sand fleas is topical application of dimeticone oils (NYDA^®^), which seal the respiratory and reproductive systems of embedded sand fleas [5,6,7,8]. However, dimeticone oils are hardly ever available to affected communities. In absence of effective treatment, people commonly turn to extracting sand fleas manually with inadequate, non-sterile instruments, like thorns or safety pins [5,9]; a painful and hazardous method which can lead to serious infections and mutilations [5,10].

Population-based studies have shown high prevalence of tungiasis, for example, 25% in rural Kenya [4], 45% in rural Nigeria [11], and 43% in Brazilian fishing villages [12], and very severe cases with hundreds of sand fleas have been described in Tanzania, Colombia, and Madagascar [2,13,14]. In Napak District, rural northeastern Uganda, our study team recently found extremely high tungiasis prevalence of 62.8% [15]. Despite its endemic presence in many countries, tungiasis has been neglected by healthcare professionals and researchers alike [1,16].

Neglected tropical diseases (NTDs) are known to be highly stigmatized, not least because of their association with poverty [17,18,19,20,21,22]. NTDs are also understudied, and this is even more true for NTD-related stigma [18,21], with the exception of widely studied leprosy stigma [17,23,24,25,26]. Disability-adjusted life years (DALYs) and economic impact of specific NTDs have been useful to demonstrate disease burden, but more attention needs to be paid to the problem of social stigma [21].

Stigma reduces life opportunities, exposes those affected to discrimination, and negatively affects mental and physical health [27]. Health-related stigma has been described as “a social process or related personal experience characterized by exclusion, rejection, blame, or devaluation that results from experience or reasonable anticipation of an adverse social judgment about a person or group identified with a specific problem” [28] (p. 280). In relation to NTDs, stigma not only adds significant psychological suffering to physical and economic hardship, but the resulting social isolation can further trap those affected in a cycle of poverty [13,18]. Neglected diseases are, in fact, diseases of neglected people [29,30]. This calls for a bio-social approach that goes beyond drug administration and takes into account local practices and power dynamics [31], thus highlighting the social factors that contribute to continued morbidity [32].

This study aims at developing such a bio-social approach to tungiasis control and stigma in the impoverished Napak District in northeastern Uganda, where tungiasis is hyperendemic. The aim of this study was to investigate local attitudes and control practices regarding tungiasis, with a focus on stigma. We ask:

- What are people’s attitudes towards tungiasis?
- How does stigma play out when a condition is so common that most people are affected?
- How does stigma relate to local tungiasis control practices?

## 2. Materials and Methods

### 2.1. Study Design and Context

This article presents stigma-related data derived from a household-based knowledge, attitudes, and practices (KAP) survey (Supplement S1), conducted between February and September 2021.

This study took place during the initial phase of a two-year long tungiasis control intervention project (2021/2022), the results of which are published elsewhere in this issue [15]. The larger intervention aimed at eliminating tungiasis as a public health problem in the area and included regular screening of the population for tungiasis; treatment of cases with a mixture of two dimeticone oils (NYDA^®^); and tungiasis-related health education and community engagement in Napak District, Uganda.

Our study team applied a KAP questionnaire to a representative member of each household in the study area who identified as the main household caretaker. We included 1329 out of a total of 1338 households in the study area (nine did not consent). Households included in the study presented here had not been exposed to the intervention at the time of data collection, i.e., they had not previously been examined for tungiasis and had not received tungiasis-related treatment or health information from our team.

### 2.2. Study Site and Population

The study took place in 17 villages located in three of the five parishes that constitute the Ngoleriet Subcounty in Napak district, Karamoja region, northeastern Uganda. Based on the national census in 2014 and local population growth rates, the total population of Ngoleriet Subcounty in 2021 has been estimated at 13,400 [33]. This rural study area was chosen because the District Health Office had alerted our study team to the high occurrence of tungiasis in the area. In a fact-finding pilot study in November 2020, our team confirmed that tungiasis was highly prevalent in the three parishes, using rapid assessment for tungiasis [34] in 11 villages. The pilot study showed a tungiasis prevalence of 68.5% (n = 456) among 666 examined individuals [15].

The total population of the 17 villages in our study area was 5482 individuals during the project’s baseline evaluation in February/March 2021 [15]. They belong to the semi-nomadic Karamojong ethnic group living in small villages which were further compartmentalized into *manyatas*, groups of houses surrounded by stick fences as protection against wild animals and animal raiders. Karamoja has a long history of animal raids and ethnic conflict and has long been a marginalized region in Uganda with widespread poverty [35]. Houses in the study area were predominantly made of sticks with grass roofs and earthen floors, which were sometimes smeared with cow dung to harden and smoothen the surface. Living conditions in the study area were generally very poor, and hunger and malnourishment were common (unpublished observation, F.M. and M.B.). Access to water was limited, as the few existing boreholes and shared water taps were located at distances up to 3 km from people’s homes and were prone to breaking. Traditionally, Karamojong men are cattle herders [35], and only few animals were kept in the villages, while most were taken to other places for grazing and to protect them from raids. Women usually stayed in the villages when men were away herding the animals. In addition to cattle herding, small-scale crop farming and low-wage day labor were common sources of income. The local population had very limited access to formal medical care, as health units were understaffed and located far away from the villages.

### 2.3. Data Collection and Analysis

Data was collected by Village Tungiasis Health Workers (VTHW), who had been trained over seven days particularly for this study and the tungiasis intervention program. They were supported by Village Health Teams (VHT) and local village leaders who mainly worked as mobilizers. The VTHW consisted of bi-lingual, literate individuals local to the study area who communicated with the respondents in Ngakarimojong language and recorded their answers in English. They were accompanied by members of our research team (F.M. and M.B.) as well as a study nurse and a social worker who assisted them in using mobile phones to record questionnaire data in ODK collect, an open-source digital Android app.

Our team designed the KAP questionnaire specifically for the tungiasis project. It consists of 50 questions, including binary questions, questions with multiple answer options, and open-text questions. Questions with pre-defined response options were not read aloud to respondents to avoid influencing their answers. Instead, the data collector chose the response category that best fitted the given answer. Responses to open-text questions were summarized and entered into an open-text box by the data collector. The questionnaire also asked about the sociodemographic information of our respondents.

Following the questionnaire interview, VTHWs physically examined the participants for tungiasis on the feet and other potentially exposed body sites. Tungiasis cases were treated topically with dimeticone oils (NYDA^®^).

Questionnaire data and clinical data about tungiasis infection were transferred to Microsoft Excel (2016) and double-checked for consistency. For this article, we purposely selected 11 relevant questions from the KAP survey that related to stigma and tungiasis control practices (Supplementary S1). One of these questions was from the “knowledge” section of the questionnaire, four from the “attitudes” section, and six from the “practices” section. We performed statistical analysis in Microsoft Excel (2016) and SPSS (IBM SPSS Statistics Version 25). Open-text questions were thematically analyzed by clustering similar answers (coding) and defining labels for the resulting thematic categories [36]. This approach allowed us to subsequently quantify frequencies of the identified response categories.

### 2.4. Ethical Considerations

Informed written consent was taken from each study participant. The VTHWs explained the aims and methods of the study in simple words in Ngakarimojong language, and individuals had time to ask any questions. Refusal to participate in the survey did not affect the right to be treated for tungiasis. Study participants gave consent by signing a form that was read out to them or by providing their fingerprint if they could not write their name. In the case of minors (under 18 years of age), both the minor and an adult caretaker were asked for informed written consent. The questionnaire interviews and physical examinations were conducted in a place chosen by the respondent.

Ethical approval for this study and the intervention program was given by the Vector Control Division of the Ugandan Ministry of Health Ethical Committee (Ref: VCDREC112/UG-REC-018) and the Uganda National Council of Science and Technology (Ref: HS2623).

## 3. Results

Our 1329 questionnaire respondents represented their households as the family members who took on most of the caring responsibilities in the household. Sociodemographic characteristics are presented in Table 1. Our respondents were mostly women (89.4%), and the median age of the respondents was 44 years (min 9 years/max 115 years). Respondents most frequently described their main occupation as casual labor (43.2%) or “none” (29.1%); and some as small-scale crop farming (14.9%); small business (9.1%); and other occupations (3.7%) that include students, employees, and others. Formal education levels among our respondents were extremely low; the vast majority (84.7%) had never attended school (Table 1).

The prevalence of tungiasis among our respondents was very high. Physical examination showed that tungiasis was present in 811 (61.0%; 95%CI 58.3–63.7%) of our 1329 respondents. Among the respondents with tungiasis, the median number of lesions was 14 (min/max: 1/591; IQR 23).

Tungiasis was not only very common but was also perceived as a potentially serious and debilitating condition. When asked if tungiasis could cause severe illness, almost all respondents (*n* = 1293/1329; 97.3%) answered “yes”. When asked if tungiasis affected their everyday life, over half of the respondents (*n* = 734/1329; 55.2%) said “yes”, referring to their own and/or their family members’ tungiasis infection. Those who had answered “yes” were asked how tungiasis affected people’s lives. The most frequently described impact was difficulty to walk and work, followed by loss of appetite and weight, fear of death, and isolation/social problems (Table 2).

### 3.1. Embarrassment

Despite the very high prevalence of tungiasis in the community, over half of the 1329 respondents (*n* = 719; 54.1%) said they felt embarrassed when having sand fleas. Examples for reported reasons for tungiasis-related embarrassment are: “[Tungiasis] brings public shame and isolation”; “They will talk and laugh about people with jiggers”; and “People begin abusing because it shows that you are not responsible towards yourself”. Most frequently, responses referred to feelings of shame, fear of social isolation and stigmatization, severe pain, being ridiculed/talked about/abused, intense itching, and fear of dying (Table 3). It should be noted that these categories are not well-distinguished from each other. The category “feelings of shame”, for example, may include all the other listed reasons for embarrassment, and “fear of social isolation and stigmatization” may also refer to, for example, the result of “being ridiculed, talked about, and abused” because of severe pain or itching that was difficult to hide. Table 3 thus displays various interconnected aspects of tungiasis-related embarrassment. These responses demonstrate that even in a community with a very high prevalence of tungiasis, embarrassment around the condition persists.

### 3.2. Association of Tungiasis with Lack of Hygiene

The KAP questionnaire further revealed that respondents frequently associated tungiasis with dirty homes and lack of bodily hygiene. When asked to name different factors that increased the chances of an individual to contract tungiasis, the most frequent answers were dirty/dusty floor in the house, mentioned by 86.5% of respondents (*n* = 1150); poor bodily hygiene, named by 70.5% of respondents (*n* = 937); and poor housing, specified by 59.7% of respondents (*n* = 793). In accord with the notion that lack of hygiene caused tungiasis, the most frequently named methods for tungiasis control were regular washing of the feet, named by 90.4% of respondents (*n* = 1202), and keeping the houses/compounds clean, mentioned by 76.2% of respondents (*n* = 1013). In short, our respondents perceived failure of keeping one’s body and home clean as the most important risk factors for tungiasis.

### 3.3. Attitudes towards People with Tungiasis

Our field team asked the respondents “What do you think about people with tungiasis?”, and coding of the responses resulted in the response categories shown in Table 4. We grouped these categories as “judging attitudes”, “compassionate attitudes”, and “other comments”. “Judging attitudes” were displayed by 42.0% of our respondents and included characterizations of people with tungiasis as lazy, careless, dirty, irresponsible, and drunkards. Compassionate attitudes were expressed by 36.3% of our respondents. They included the representation of people with tungiasis as in need of help, being very sick, being elderly or disabled, being people too, as well as expressions of sympathy, such as “I feel sorry for them”. Other comments could not clearly be labelled as either judging or compassionate. These included the view that people with tungiasis needed more advice on how to prevent or treat sand fleas, and other statements (Table 4).

Judging attitudes were very widespread despite the very high prevalence in the community and the fact that most respondents had tungiasis themselves at the time of data collection. Members of our field team (F.M. and M.B.) clarified that having a few sand fleas was seen as normal in the villages, and that respondents will most likely have referred to severe cases of tungiasis in their responses. To investigate the stigmatization of heavy tungiasis infection further, we separately analyzed responses of individuals with 30 or more tungiasis lesions on the body, defined as heavy tungiasis infection [37]. At the point of data collection, 193 (14.5%) of our 1329 respondents had heavy tungiasis infection. As expected, they expressed more compassionate attitudes (52.9%; *n* = 102), primarily sympathy (21.2%; *n* = 41) and the need for help (16.1%; n = 31). However, even among the heavily affected, 21.2% (*n* = 41) displayed judging attitudes by labelling people with tungiasis as lazy (8.8%; *n* = 17), careless (6.2%; *n* = 12), irresponsible (4.1%; *n* = 8), and dirty (2.1%; *n* = 4).

### 3.4. Treatment and Prevention Practices

When asked how they treated tungiasis in their families, 84.1% (*n* = 1119) of respondents named extraction with sharp instruments like thorns, needles, pins, and razor blades, and 14.8% (*n* = 197) said they applied various substances (Table 5), mainly greasy products like petroleum jelly. When asked about additional treatment practices, 74 (5.6%) mentioned manual extraction of sand fleas, which raises the reported prevalence of this practice to 89.7% (*n* = 1193).

Those who had stated manual extraction of sand fleas as the main treatment (n = 1119) were asked about details of their extraction practice (Table 6). The majority (63.8%) said they shared their extraction instruments with others, and 53.1% said they boiled them. Use of antiseptics was reported by 14.3%, and 58.4% (*n* = 654) named one or several substances they applied to the wound after extraction. Hot or cold ash (*n* = 342) and tobacco (*n* = 296) were mentioned most often, together with greasy substances (*n* = 67) like petroleum jelly. Some also mentioned cooking oil, castor oil, paraffin, and harmful substances like used engine oil, diesel, and petrol. These substances were often applied as mixtures, for example cooking oil mixed with tobacco and ash. Furthermore, 25 respondents named herbal remedies, including *aloe vera*, milk bush sap, balamite, a local tree called *epuu*, a fruit called *eome*, seeds from the *ekolej* tree, and others.

In the study area, the houses had earthen floors. People habitually walked barefoot or used rubber sandals. The great majority of respondents (93.8%) stated that they washed their feet once or several times per day (Table 7). Similarly, most respondents reported that they swept their houses and their compounds daily (83.5% and 76.1, respectively). Only a small minority said they swept their houses and their compounds less than every other day (4.8% and 10.8%, respectively).

In addition to sweeping, 260 respondents (19.6%) said they had applied commercial insecticides in their houses in the past, namely the toxic substances “Dudu Dust” (carbaryl) [38] and “Supona” (chlorfenviphos) [39]. However, our field team observed that insecticides were rarely available (unpublished observation, F.M. and M.B.). Several respondents also mentioned smearing house floors with cow dung as a traditional way of keeping them smooth and clean, but as most cattle was herded away from the villages, cow dung was scarce (unpublished observation, F.M. and M.B.).

## 4. Discussion

### 4.1. Attitudes towards Tungiasis

Our finding that most respondents perceived tungiasis as a potentially serious and debilitating condition contrasts with the reported perception that tungiasis is not an important health threat in affected communities in Northeast Brazil [16]. However, the strong impact of tungiasis infection on everyday life in our study is consistent with findings from a study on children in Nigeria, where 78% of affected children reported that tungiasis had a moderate or severe effect on their quality of life, which rapidly improved after effective treatment [4]. Impairments reported by our respondents, namely mobility restrictions, sleep disturbances, pain and itching, social isolation, and being ridiculed have been described for tungiasis before [40,41]. Similar problems have also been reported regarding other NTDs, like cutaneous *larva migrans* [42,43], lymphatic filariasis [44], and leprosy [25]. However, our finding that some respondents associated tungiasis with weight loss and fear of dying is remarkable and highlights the severity of the problem in Napak District. Considering that the local population lives in dire poverty [45], with hunger and malnutrition being commonplace, a parasitic infection like tungiasis can quickly become a physically and socially existential threat. This finding is corroborated by a series of extremely severe tungiasis cases in indigenous villages in the Colombian Amazon region [13], where affected individuals presented with life-threatening malnutrition, severe anemia, weight loss, and immobility and were left by their families to die in the forest.

Our study further provides insights into tungiasis-related stigma by presenting widespread judging attitudes and stereotyping labels, such as “lazy”, “careless”, “dirty”, and “irresponsible”. Similarly, a study from rural Eastern Uganda reported that 31% of their respondents characterized individuals/families with tungiasis as “lazy” and 17% as “irresponsible” [46]. However, in contrast to our findings, this study did not mention an association with dirtiness. “Dirty” is a common stigma label that particularly affects individuals and groups with poverty-associated conditions [47]. In a qualitative study in Kenya, tungiasis sufferers felt that they were perceived as dirty and disgusting in the community [48]. According to labelling theory in stigma research, the judging labels we identified are “informal labels” that affect individuals in day-to-day interactions in their community [49]. Stigma has famously been described as an “attribute that is deeply discrediting”, marking a person as a “tainted, discounted one” [50] (p. 3). Stigma thus contributes to significant suffering and an often hidden burden of illness [28], and NTDs are known to be particularly highly stigmatized conditions due to their common association with poverty, physical impairment, and disfigurement [17,21]. However, stigma usually affects minority groups who are “distinguished […] as a separate social entity” [51] (p. 462). The finding that tungiasis-associated stigma was still highly relevant in a community where the *majority* was affected thus deserves further attention.

### 4.2. Stigma in a Hyperendemic Environment

The high prevalence of tungiasis infection (61.0%) among the interviewed household caretakers corresponds with the prevalence of 62.8% among the overall population in our study area in Napak district, northeastern Uganda [15]. Tungiasis is known to be endemic in Uganda, especially in rural communities in Eastern regions, where a study found that in 22.5% of households, tungiasis infection was present in at least one household member [46]. Population-based studies from Kenya, Nigeria, and Brazil show high tungiasis prevalences of 25 to 56% [4,11,12,52]. However, communities with very high prevalences of over 60%, like in Napak district, have—to our knowledge—not been studied before.

In the studied setting, where single tungiasis lesions were seen as normal (F.M. and M.B., unpublished observation), stigmatization did not affect everyone with tungiasis in the same way. We can expect a gradual degree of stigmatization and that the judging and compassionate attitudes, together with the respective labels, more greatly affect those who bear the most notable signs of tungiasis. Similarly, a study of lymphatic-filariasis-associated stigma in Nigeria [53] showed that those with the most pronounced signs of the disease were most stigmatized.

Interestingly, judging attitudes towards people with tungiasis were also expressed by respondents who were themselves heavily infected, albeit only half as often as in the overall study population (21.2% vs. 42%). This finding might be linked to an attempt to distance oneself from the stigmatized group, in itself an expression of “felt stigma”, with its two components of feeling shame and fear of discrimination [54]. Furthermore, the expressed judgmental attitudes might indicate a degree of “self-stigma”, the internalization of normative stigma by those who possess stigma markers. In cases of self-stigma, “people’s self-concept is congruent with the stigmatizing responses of others; they accept the discredited status as valid” [55] (p. 3).

### 4.3. Stigma and Tungiasis Control Practices

The vast majority of our respondents (89.7%) stated that they practiced manual extraction of sand fleas with sharp instruments, which is known as a common method in endemic communities [5,9,10]. Manual extraction is painful and dangerous as it can lead to mutilations and life-threatening bacterial infections [5,56], but in the described context, people had few alternatives. The application of hazardous substances to the affected skin (like tobacco and engine oil), which some of our respondents described, can lead to skin damage and intoxication and has been described for other NTDs in areas with limited access to medical care, for example cutaneous *larva migrans* in Brazil [43].

Importantly, the presented treatment and prevention practices (including hygiene measures) require various resources: water and soap to wash the feet; mobility to sweep the house and compound every day; good eyesight and fine motor skills to perform manual extraction of sand fleas; money to buy substances to apply to lesions; and the ability to prioritize hygiene and self-care in a setting where hunger and scarcity are commonplace [45]. As embedded sand fleas continually shed eggs onto the ground [3], people with the least individual and social resources who cannot keep their houses and bodies clean and remove sand fleas from their feet, will accumulate eggs and larvae in their immediate environment and hence be vulnerable to repeated and severe tungiasis infections [4]. We can assume that this will lead to a vicious cycle in which they become more and more immobilized and dependent on help from others, while at the same time they are also increasingly at risk of being stigmatized and socially isolated.

Our finding that some of the interviewed household caretakers stated that people with tungiasis “need advice” (8.0%; n = 106) suggests that tungiasis-related community sensitization might be useful to support tungiasis control in this setting. Indeed, community engagement could build on the finding that 36.3% of our respondents expressed compassionate attitudes towards people with tungiasis, such as “they need help”, which indicates the presence of—or at least potential for—networks of care within the community. However, health education may have unintended consequences. Anthropologists argue that global health campaigns that foreground hygiene education and implementation often inadvertently increase “hygiene stigma” in the community, which causes further shame and isolation for those who are unable to put the set hygiene goals into practice [47]. When this happens, people might resort to manual extraction of sand fleas even more to avoid being seen as dirty.

Whilst Stigmatizing, both the common association of tungiasis with dirtiness and the social isolation of patients are, from a medical perspective, reasonable. Lack of personal hygiene and dirty/dusty floors have indeed been established as risk factors for tungiasis [4,37]. As sand fleas embedded in the skin continuously excrete eggs onto the ground [57,58], the infested soil in places where affected individuals reside or walk poses a risk of infection to others. This consideration, however, contrasts with the definition of health-related stigma as “*medically unwarranted* with respect to the health problem itself” [28] (p. 280, our emphasis). Although attributes like “lazy”, “careless”, and “dirty” carry unwarranted moral judgment about tungiasis-affected individuals, their social isolation can (at least partly) be understood as a *medically warranted* attempt to avoid spreading and acquiring tungiasis infection in the absence of effective and safe treatment. Health education alone is therefore unlikely to reduce stigmatization.

Access to safe and effective tungiasis treatment would make painful and dangerous treatment attempts obsolete and would presumably lower the burden of stigma and social isolation significantly. However, we recognize that “[m]ore medicines alone cannot ensure the treatment of neglected tropical diseases” [59] (p. e330). Integration of treatment into local health systems, possibly at a village level, is necessary to make it accessible to those who need it most and to ensure the sustainability of treatment success. Community engagement is an essential tool to establish understanding of treatment and prevention and build local networks of care, ideally with formalized and paid community health workers [60].

### 4.4. Study Limitations

We included a small number of children under 16 years of age in this study (n = 14; minimum age = 9 years), as in rare cases children lived in their own huts near other family members’ accommodation. However, the questions were simple so that we could assume that the children were able to answer them appropriately. Twelve respondents reported they were over 90 years old (maximum age = 115 years), which in the presented context may simply mean “very old”, as not everyone knows their exact age. Similarly, precise information about the respondents’ main occupation was difficult to obtain as people had little to no school education and no formal employment, and they used various strategies at the same time to make ends meet.

The KAP questionnaire was developed based on the expertise of our team members and had not been previously used or pre-tested. Self-reported information about prevention and treatment practices is prone to information bias, and we did not observe if our respondents acted according to their claims. In particular, hygiene measures (frequency of cleaning/washing, boiling of extraction instruments, and use of antiseptics before manual extraction) may have been overstated.

## 5. Conclusions

Despite the high prevalence of tungiasis in Napak District, stigma and shame around the condition were common. Judging attitudes about people with tungiasis were widespread and could partly be attributed to the commonly made association of tungiasis with dirtiness. While hygiene measures are a key aspect of tungiasis control, prioritising hygiene education thus bears the risk of further increasing pre-existing “hygiene stigma”, especially when water is scarce. The dangerous and painful method of extracting sand fleas with sharp instruments was very common. Making safe and effective treatment available in the community can be expected to make dangerous treatment attempts obsolete and to break the vicious cycle of tungiasis infection, impairment, stigma, and social isolation.

## Figures and Tables

**Table 1:** Sociodemographic characteristics of the 1329 questionnaire respondents.

Socio-Demographic Characteristics	Number	%
**Gender**		
Female	1188	89.4
Male	141	10.6
TOTAL	1329	100
**Age group**		
<20 years	51	3.8
20–39 years	536	40.3
40–59 years	336	25.3
>60 years	406	30.5
TOTAL	1329	100
**Occupation**		
Casual laborer	574	43.2
Small-scale crop farming	198	14.9
Small business	121	9.8
None	387	29.1
Other	49	3.7
TOTAL	1329	100
**Formal education (highest level)**		
None	1126	84.7
Some primary/completed primary school	173	13.0
Some secondary/completed secondary school	30	2.3
TOTAL	1329	100

**Table 2:** Effects of tungiasis on everyday life (open-text question). Several individuals gave more than one response; therefore, the number of responses (*n* = 755) was higher than the number of respondents (*n* = 734).

Effects of Tungiasis on Daily Life	Number of Responses	% of Responses
Difficulty to walk and work	348	46.1
Loss of appetite and weight	141	18.7
Fear of dying	65	8.6
Isolation/social problems	69	9.1
Pain and itching	26	3.4
Sleeplessness	21	2.8
Other problems (incl. physical deformation, stress, and anemia)	85	11.3
TOTAL	755	100

**Table 3:** Reasons for feeling embarrassed of tungiasis (open-text question).

Reasons for Embarrassment	Number	%
Feelings of shame	133	18.5
Fear of social isolation/stigmatization	100	13.9
Severe pain	90	12.5
Being ridiculed, talked about, and abused	82	11.4
Intense itching	76	10.6
Fear of dying	41	5.7
Wounds, swelling, and foot deformations	33	4.6
Others being fearful of them	21	2.9
Sleeping problems	20	2.8
Other	123	17.1
TOTAL	719	100

**Table 4:** Judging and compassionate attitudes towards people with tungiasis (open-text question).

Attitudes	Number (*n* = 1329)	%
**Judging attitudes**		
They are lazy	188	14.1
They are careless	164	12.3
They are dirty	99	7.4
They are irresponsible	90	6.8
They are drunkards	17	1.3
TOTAL	558	42.0
**Compassionate attitudes**		
They need help	175	13.2
They are very sick	135	10.1
I feel sorry for them	120	9.0
They are elderly or disabled	34	2.6
They are people, too	19	1.4
TOTAL	483	36.3
**Other comments**		
They need advice	106	8.0
Nothing/none of my business	80	6.0
Other	75	5.6
I fear getting infected	27	2.0
TOTAL	288	21.7

**Table 5:** Treatment practices for tungiasis. The question about the main practice offered several response options. The question about additional practices was answered as open-text.

Treatment Practices	Number (*n* = 1329)	%
**Main practice**		
Manual extraction	1119	84.1
Application of various substances	191	14.4
Other	10	0.8
I don’t know/Nothing	9	0.7
**Additional treatment practice**		
Manual extraction	74	5.6
Application of various substances	59	4.4

**Table 6:** Details about manual extraction practice. These questions were only asked if manual extraction was stated as main treatment method (*n* = 1119).

Manual Extraction Practice	Number (*n* = 1119)	%
Use of shared instruments	714	63.8
Use of boiled instruments	594	53.1
Application of antiseptic before extraction	160	14.3
Application of substances to the wound after extraction	192	17.2

**Table 7:** Reported frequencies of cleaning the feet, house, and compound.

Preventive Measure (Frequency)	Number	%
**Washing feet**		
Several times per day	707	53.2
Once per day	539	40.6
Every other day	35	2.6
Fewer times	48	3.6
TOTAL	1329	100
**Sweeping house**		
Every day	1110	83.5
Every other day	155	11.7
Fewer times	64	4.8
TOTAL	1329	100
**Sweeping compound**		
Every day	1011	76.1
Every other day	175	13.2
Fewer times	143	10.8
TOTAL	1329	100

## Data Availability

The data presented in this study are available upon request from the corresponding author. The data are not publicly available due to the sensitive nature of the information, including statements about stigmatization, and the slight risk that individuals might be identified through their sociodemographic details and location.

## Supplementary Materials

The following supporting information can be downloaded at: https://www.mdpi.com/article/10.3390/tropicalmed8040206/s1. S1. Selected Questions from KAP Questionnaire.
