# Supplementary material for: Tungiasis Stigma and Control Practices in a Hyperendemic Region in Northeastern Uganda
Source: Trop Med Infect Dis. 2023 Mar 30;8(4):206. doi: 10.3390/tropicalmed8040206 (PMC10144114; doi:10.3390/tropicalmed8040206)
Supplement: Supplementary file 1 [file tropicalmed-08-00206-s001.zip › tropicalmed-2314766-supplementary.pdf]

Supplement S1: Selected Questions from KAP Questionnaire

KNOWLEDGE

Name at least four risk factors (factors which increase the chances) for getting jiggers.

(Response options: 1=dry/dusty/dirty floor; 2=poor housing; 3=poor body hygiene; 4=living/working with animals; 5= no footwear; 6=dry weather conditions; 7=sleeping on the floor; 8=crowded homes; 9=open defecation; 10=disposal of waste on the compound; 87= other)

ATTITUDES

What do you think about people with jiggers? (Open text response)

Do you feel embarrassed when finding jiggers in your feet? (Y/N)

- If yes, why? (Open text response)

Do you think jiggers can cause severe disease? (Y/N)

Does having jiggers affect one's every day life? (Y/N)

- If yes, how? (Open text response)

PRACTICES

How often do you wash your feet? (Response options: Several times per day / Once per day / Every other day / Fewer times)

How often do you sweep your house? (Response options: Every day / Every other day / Fewer times)

How often do you sweep the compound? (Response options: Every day / Every other day / Fewer times)

How do you treat jiggers in your family? (Response options: Extraction with sharp instruments / Application of agrochemical substances / Application of BBE / I don't know / Other)

Do you use any other treatment? (Open text box)

If you treat by extraction,

- do you boil the extraction instrument? (Y/N)
- do you apply an antiseptic before extraction? (Y/N)
- do you share the instrument with other people? (Y/N)
- do you apply anything to the wound? (Y/N)
    - if yes, what do you apply? (Open text response)
